# Relevance of concurrent hypercalcemia in ureteric sarcoidosis complicated with bladder urothelial carcinoma: a case report

**DOI:** 10.1186/s12882-020-01893-8

**Published:** 2020-06-22

**Authors:** Michikata Hayashida, Akihiro Yano, Kiichi Hagiwara, Shoichi Nagamoto, Kohei Ogawa, Kazushige Sakaguchi, Naoki Sawa, Toshikazu Okaneya, Shinji Urakami

**Affiliations:** 1 Department of Urology, Toranomon Hospital, 2–2–2 Toranomon, Minato-ku, Tokyo, 105–0001 Japan; 2 Department of Nephrology, Toranomon Hospital, Tokyo, Japan

**Keywords:** Ureteral cancer, Ureteric sarcoidosis, Bladder urothelial carcinoma, Hypercalcemia

## Abstract

**Background:**

Sarcoidosis is a multisystem inflammatory disorder and can affect any organ; however, ureteric involvement is extremely rare with only four cases reported in the literature to date, all of which were diagnosed with surgical ureteral resection including a nephroureterectomy. This study reports the first case of ureteric sarcoidosis controlled with medical therapy where a differential diagnosis was performed based on the diagnostic clue of hypercalcemia. A definitive diagnosis was established without surgical resection of the ureter.

**Case presentation:**

A 60-year-old man presented with anorexia and weight loss. Blood tests showed renal dysfunction and hypercalcemia. Computed tomography revealed left hydronephrosis associated with left lower ureteral wall thickening, which showed high signal intensity on diffusion-weighted magnetic resonance imaging. Similarly, we detected a bladder tumor on cystoscopy, and a 2-cm-long stenosis was revealed by retrograde ureterography; therefore, ureteral cancer was suspected. Meanwhile, considering the clinical implication of hypercalcemia, a differential diagnosis of sarcoidosis was established based on elevated levels of sarcoidosis markers. Fluorodeoxyglucose positron emission tomography showed fluorodeoxyglucose accumulation in the left lower ureter, skin, and muscles, suggestive of ureteric sarcoidosis with systemic sarcoid nodules. For a definitive diagnosis, transurethral resection of the bladder tumor and ureteroscopic biopsy were performed. Histopathological examination revealed ureteric sarcoidosis with bladder urothelial carcinoma. Following an oral administration of prednisolone, hypercalcemia instantly resolved, the renal function immediately improved, and the left ureteral lesion showed complete resolution with no recurrence.

**Conclusions:**

In this case, the co-occurrence of ureteral lesion with bladder tumor evoked a diagnosis of ureteral cancer. However, considering a case of ureteral lesion complicated with hypercalcemia, assessment for differential diagnosis was performed based on the calcium metabolism and sarcoidosis markers. In cases of suspected ureteric sarcoidosis from the assessment, pathological evaluation with ureteroscopic biopsy should be performed to avoid nephroureterectomy.

## Background

Sarcoidosis is a multisystem inflammatory disorder characterized by the formation of non-caseating granulomas and can affect any organ [1]. However, ureteric involvement is extremely rare, with only four cases reported in the literature to date, all of which were diagnosed with surgical ureteral resection including a nephroureterectomy [2–5]. To our knowledge, this is the first case of ureteric sarcoidosis controlled with medical therapy, where differential diagnosis was performed based on the diagnostic clue of hypercalcemia, and a definitive diagnosis was established without surgical resection.

## Case presentation

A 60-year-old man with no medical history or comorbidities presented with anorexia and weight loss (from 59.4 kg before admission to 55.9 kg on admission). No findings were observed on physical examination. Blood tests showed renal dysfunction (creatinine, 2.27 mg/dL) and hypercalcemia (total serum calcium corrected for albumin, 12.2 mg/dL). A urine test revealed hypercalciuria without proteinuria (Table 1).

**Table 1:** Results of biochemical and urine analyses before and after steroid treatment

Parameter	Before steroid treatment	21 days after steroid treatment	42 days after steroid treatment
Creatinine (mg/dL)	2.27	1.24	1.32
Serum calcium^a^ (mg/dL)	12.2	9.4	9.7
1,25-(OH)_2_D_3_ (ng/L)	151	31	–
ACE (U/L)	39.7	21.4	9.6
Lysozyme (mg/L)	37.9	12.7	5.2
SIL-2R (U/mL)	3190	873	402
25(OH)D (ng/mL)	23.3	–	–
PTH (pg/mL)	15	–	–
PTHrP (pmol/L)	< 1.1	–	–
Urine specific gravity	1.006	1.012	1.021
Urine level of calcium (mg/day)	487.5	80.6	–

^a^ Serum calcium implies the total serum calcium corrected for the albumin level

**Fig. 1:**
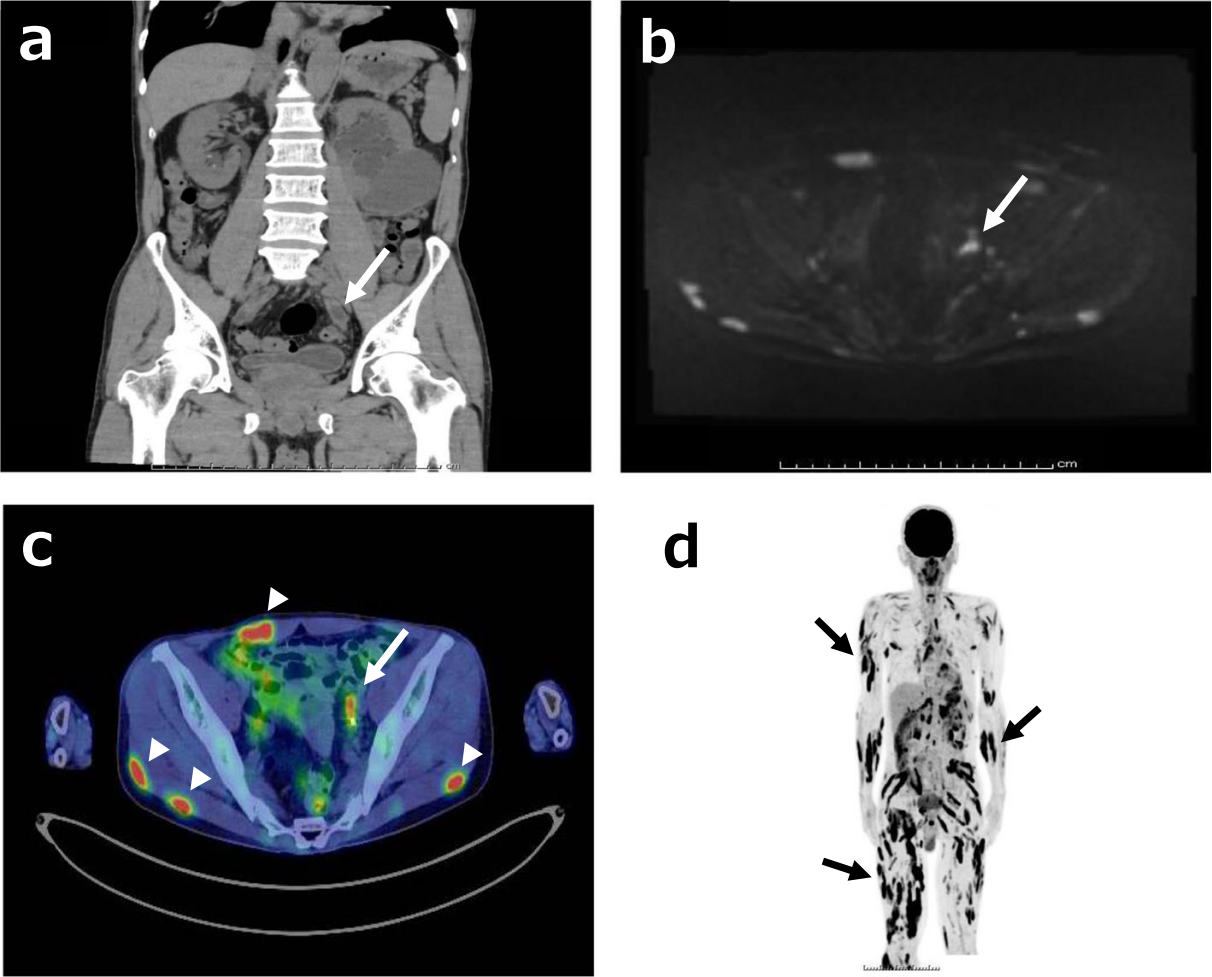
CT, MRI, and FDG-PET findings for the left lower ureteral lesion and whole-body FDG accumulation. **a.** Abdominal plain CT shows left hydronephrosis associated with left lower ureteral wall thickening (white arrow). **b** Diffusion-weighted MRI shows high signal intensity for the ureteral lesion (white arrow). **c** FDG-PET reveals FDG accumulation in the left lower ureter (white arrow), skin, and muscles (white arrowhead). **d.** The black striae formed by FDG accumulation represent sarcoid nodules in the skin and muscles. The black arrows illustrate representative sites of the sarcoid nodules. CT: computed tomography, MRI: magnetic resonance imaging, FDG: fluorodeoxyglucose, FDG-PET: fluorodeoxyglucose positron emission tomography

The hypercalcemia was treated with the administration of elcatonin and normal saline infusion for fluid replacement. Computed tomography (CT) revealed left hydronephrosis associated with left lower ureteral wall thickening, which showed high signal intensity on diffusion-weighted magnetic resonance imaging (Figs. 1a, b, 4a). Although the voided urinary cytology result was negative, a 5-mm papillary pedunculated tumor was detected on the left lateral wall of the bladder on cystoscopy; in addition, a 2-cm-long stenosis in the left lower ureter was revealed by retrograde ureterography. Therefore, a ureteral cancerous lesion was suspected.

**Fig. 2:**
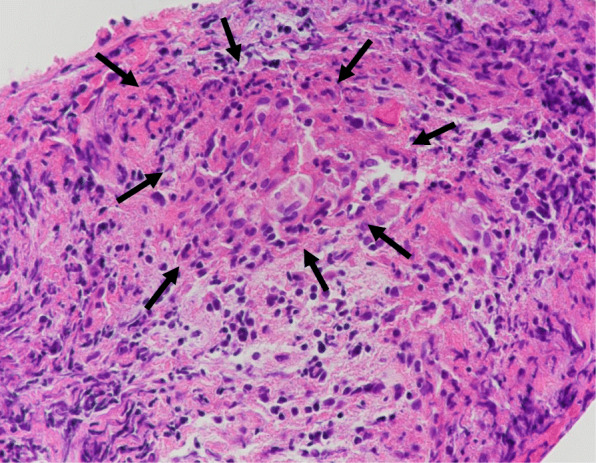
Hematoxylin and eosin staining of the left lower ureteral specimen obtained by biopsy. The arrows indicate a non-caseating granuloma

**Fig. 3:**
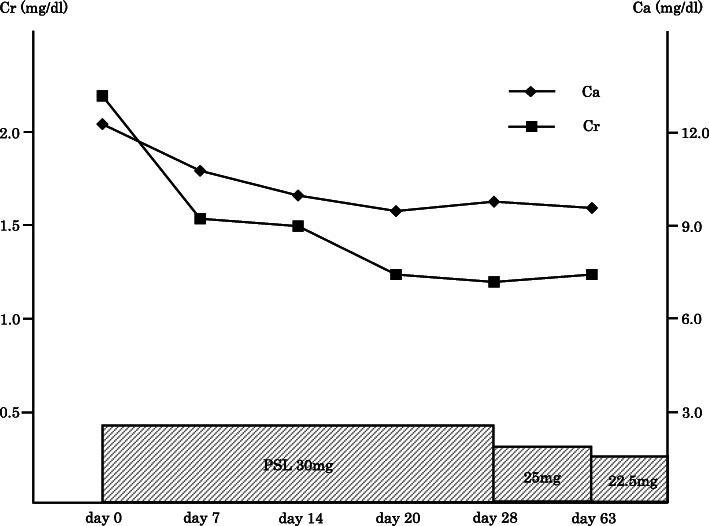
Recovery of serum calcium (corrected for albumin) and creatinine levels. Serum calcium and creatinine levels show immediate recovery after an oral administration of prednisolone. Ca: calcium, Cr: creatinine

**Fig. 4:**
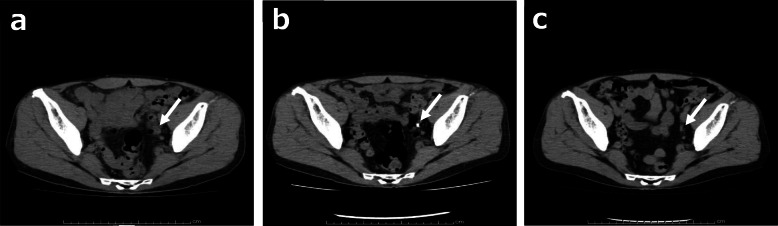
CT findings for the ureteral lesion. The white arrows indicate the left ureteral lesion. **a.** Before administration of PSL. **b**. Eighteen days after the initiation of PSL, complete resolution of the left ureteral lesion was recognized. **c**. Sixty-three days after the initiation of PSL, there was no recurrence after the removal of DJS. CT: computed tomography, PSL: prednisolone, DJS: double J stent

Meanwhile, considering the clinical implication of hypercalcemia, a differential diagnosis of sarcoidosis was established based on elevated levels of 1,25-dihydroxyvitamin D_3_ (1,25-(OH)_2_D_3_, 151.0 ng/L, normal range: 20.0–60.0 ng/L) and sarcoidosis markers: angiotensin-converting enzyme (ACE, 39.7 U/L, normal range: 7.0–25.0 U/L), lysozyme (37.9 mg/L, normal range: 5.0–10.2 mg/L), and soluble interleukin-2 receptor (SIL-2R, 3190 U/ml, normal range: 145–519 U/ml). Neither 25-hydroxyvitamin D (25(OH)D), parathyroid hormone (PTH), nor parathyroid hormone-related peptide (PTHrP) was elevated: levels of 25(OH)D, PTH and PTHrP were 23.3 (normal range: > 20.0 ng/mL) ng/mL, 15.0 (normal range: 15.0–65.0 pg/mL) pg/mL and <  1.1 (normal range: < 1.1 pmol/L) pmol/L, respectively (Table 1). Fluorodeoxyglucose (FDG) positron emission tomography (FDG-PET) showed FDG accumulation not only in the left lower ureter but also in the skin and muscles, suggestive of ureteric sarcoidosis with systemic sarcoid nodules (Fig. 1c, d). Among the sites of FDG accumulation in the skin and muscles, the nodules were palpated at the right side chest, right thigh, and right lumbar region. For a definitive diagnosis, transurethral resection of the bladder tumor (TURBT) and ureteroscopy were performed. Histopathological examination of TURBT specimens revealed pTa high-grade bladder urothelial carcinoma, with ureteroscopic evaluation showing circumferential stenosis in the left lower ureter without an obvious mass lesion. Furthermore, we selectively collected samples of left ureteral urine for cytology, performed a biopsy at the stenosis site, and subsequently placed a double -J stent (DJS). Left hydronephrosis resolved after the alleviation of the left upper urinary tract obstruction by the DJS; however, no improvement in renal function was observed. The ureteral urinary cytology result was negative, and the histopathological examination of ureteral biopsy specimens revealed non-caseating granulomas without cancerous tissue (Fig. 2), thereby confirming the diagnosis of ureteric sarcoidosis. Hypercalcemia instantly resolved, and the renal function immediately improved following an oral administration of prednisolone (PSL) (Fig. 3). Moreover, hypercalciuria diminished, and both 1,25-(OH)_2_D_3_ and sarcoidosis markers were normalized (Table 1). Eighteen days after the initiation of PSL treatment, CT showed complete resolution of the left ureteral lesion (Fig. 4b), and subsequently, the DJS was removed. Sixty-three days after the initiation of PSL, CT revealed no recurrence of the left ureteral lesion (Fig. 4c). To date, there has been no recurrence of the left ureteral lesion, hypercalcemia, and renal dysfunction in the patient who is presently on tapered PSL dose.

## Discussion and conclusions

Sarcoidosis is a multisystem inflammatory disorder of unknown etiology, characterized by the formation of non-caseating granulomas. Exaggerated immune response to an undefined antigen (e.g., environmental factors, microbes, or degraded antigen) has been implicated as a potential cause of sarcoidosis [1]. Sarcoidosis can affect any organ: the most commonly affected organ is the lungs [1]. In addition, bilateral hilar lymphadenopathy, which was not recognized in the present case, is the most common radiological finding in sarcoidosis [6]. On the contrary, ureteric involvement is extremely rare, and diagnosis of ureteric sarcoidosis before surgical resection is challenging [2–5]. In this case, clarifying the cause of hypercalcemia served as a helpful clue to the diagnosis of ureteric sarcoidosis without the need for surgical ureteral resection.

Calcium metabolism disorder, characterized by hypercalcemia and hypercalciuria, which were recognized in this case, is a clinical feature in sarcoidosis. Hypercalcemia and hypercalciuria occur in approximately 5% and 40–62% of sarcoidosis patients, respectively [7]. An increase in the production of 1,25-(OH)_2_D_3_ by non-caseating granulomas has been implicated in calcium metabolism disorder. An elevated level of 1,25-(OH)_2_D_3_ enhances intestinal calcium absorption and promotes osteoclastic activity and bone resorption, resulting in hypercalcemia and hypercalciuria [8–10].

Calcium metabolism disorder is the most important cause of renal dysfunction. Several different mechanisms, including vasoconstriction of arterioles, acute tubular necrosis, impairment of urinary concentrating ability caused by decreased sensitivity to antidiuretic hormone, and nephrocalcinosis, have been implicated in renal dysfunction [10]. In the present case, improvement in renal function was not observed after the resolution of the left upper urinary tract obstruction with DJS placement; however, renal function improved after amelioration of hypercalcemia by PSL administration. These findings suggested that sarcoidosis-related calcium metabolism disorder was the main cause of renal dysfunction, although renal biopsy was not performed.

The present case met the diagnostic criteria of sarcoidosis: a compatible clinical and/or radiological image, histopathologic evidence of non-caseating granulomas, and exclusion of other diseases with similar findings, such as infections or malignancy [11]. The indications for the treatment of sarcoidosis have been shown to be disease-related symptoms in patients or organ dysfunction [12]. In the present case, since the patient had anorexia, weight loss and renal dysfunction, which were consistent with the indications for treatment, PSL, a corticosteroid, was orally administered. Oral corticosteroids are considered the first-line treatment in the management of sarcoidosis, and the effectiveness of PSL has been reported in sarcoidosis-related hypercalcemia [7, 10, 13]. In fact, PSL was an effective treatment for ureteric sarcoidosis with hypercalcemia in this case.

In the present case, the co-occurrence of ureteral lesion with bladder tumor evoked a diagnosis of ureteral cancer. Meanwhile, the clinical manifestation of hypercalcemia served as a clue to the differential diagnosis of ureteric sarcoidosis through the elevated levels of 1,25-(OH)_2_D_3_ and sarcoidosis markers (ACE, lysozyme, and SIL-2R). However, it should be noted that urothelial carcinoma is rarely complicated by hypercalcemia as paraneoplastic syndrome [14–16]. In this case, no increase was observed in the PTHrP level, which has been most commonly associated with paraneoplastic hypercalcemia in various malignancies [15]. In ureteral cancer, 1,25-(OH)_2_D_3_ is similarly reported to be one of the inducers of paraneoplastic hypercalcemia [16]. Therefore, the measurement of sarcoidosis markers in addition to that of calcium metabolism markers, such as PTHrP and 1,25-(OH)_2_D_3_, would be needed to clarify the cause of hypercalcemia. Finally, ureteroscopy is a useful tool, which can facilitate more precise pathological diagnosis [17]. In the present case, ureteroscopic biopsy contributed to the definitive diagnosis of ureteric sarcoidosis, avoiding surgical resection.

In conclusion, in the cases of ureteral lesions complicated with hypercalcemia, assessments for differential diagnosis based not only on calcium metabolism markers but also on sarcoidosis markers should be performed as a first step. This can help decide whether subsequent examinations for ureteric sarcoidosis are necessary. In the event ureteric sarcoidosis is suspected after the initial assessments, pathological evaluation with ureteroscopic biopsy should be performed, as ureteric sarcoidosis with hypercalcemia can be controlled with medical therapy, thus avoiding nephroureterectomy.

## Data Availability

Not applicable.

## Abbreviations

CT: Computed Tomography
1,25-(OH)_2_D_3_: 1,25-dihydroxyvitamin D3
ACE: Angiotensin-converting enzyme
SIL-2R: Soluble interleukin-2 receptor
25(OH)D: 25-hydroxyvitamin D
PTH: Parathyroid hormone
PTHrP: Parathyroid hormone-related peptide
FDG: Fluorodeoxyglucose
FDG-PET: Fluorodeoxyglucose positron emission tomography
TURBT: Transurethral resection of bladder tumor
DJS: Double J stent
PSL: Prednisolone
